# Perivascular Epithelioid Cell Tumor (PEComa) Affecting the Knee in a Seven-Year-Old Girl: A Case Report

**DOI:** 10.7759/cureus.107702

**Published:** 2026-04-25

**Authors:** Nikolaos Laliotis, Panagiotis Konstantinidis, Chrysanthos Chrysanthou, Themistoklis Konstantinidis, Anastasia Lalioti

**Affiliations:** 1 Orthopaedics, Interbalkan Medical Center, Thessaloniki, GRC; 2 Occupational Therapy, University of Western Macedonia, Kozani, GRC; 3 Pathology, Interbalkan Medical Center, Thessaloniki, GRC; 4 Medical Oncology and Hematology, Humanitas Research Hospital, Milan, ITA; 5 Biomedical Sciences, Humanitas University, Milan, ITA

**Keywords:** benign tumor, knee region, mesenchymal tumors, pecoma, perivascular epithelioid tumors

## Abstract

Perivascular epithelioid cell tumors (PEComas) are rare mesenchymal tumors composed of cells with epithelioid and spindle morphology, which grow around blood vessels. PEComas of soft tissue are usually reported in adults, with a rare incidence in the pediatric population, especially in young children and in the soft tissue of the limb. We report a case involving a healthy seven-year-old girl who presented with a painless prominence in front of the tibial tubercle. Radiological imaging revealed an encapsulated, lobulated mass that simulated a lipoma. After surgical excision, the microscopic examination and histopathological analysis of the surgical specimen confirmed the diagnosis of benign PEComa. Our case underscores the importance of considering PEComa in the differential diagnosis of pediatric soft tissue masses in children, whose presentation may vary from benign to malignant. Surgical treatment is the preferred approach for benign PEComas.

## Introduction

Perivascular epithelioid cell tumors (PEComas) constitute a rare family of mesenchymal neoplasms characterized by a unique morphologic and immunophenotypic profile, composed of spindled to epithelioid cells that co-express both melanocytic and myogenic markers. While these tumors are predominantly diagnosed in the adult population, pediatric occurrences - particularly those involving the soft tissue - remain exceedingly rare. PEComas exhibit a broad anatomic distribution, including the uterus, kidneys, liver, lungs, gastrointestinal tract, and pancreas, with soft tissue involvement of the limbs being particularly uncommon [1-5].

Some PEComas may be associated with genetic syndromes, such as tuberous sclerosis complex (TSC). A case of a four-year-old boy with TSC presenting a fibrin-like PEComa has been reported [6]. In children, PEComas more frequently affect the gastrointestinal tract. A PEComa affecting the thigh was reported in an 11-year-old girl [3,7].

We present a unique case of a healthy seven-year-old girl who exhibited a painless swelling in the prepatellar tendon area of the right knee. Initial radiological investigations, including X-rays and magnetic resonance imaging (MRI), revealed a well-defined soft tissue mass that was encapsulated, with no fluid or hemorrhagic elements and without bone involvement. An initial diagnosis of a lipoma was suspected. The mass was surgically excised, and histopathological examination revealed an architecture of large epithelioid and spindle cells interspersed with numerous thin-walled vessels. The tumor cells were positive for vimentin and exhibited focal positivity for desmin, confirming the diagnosis of PEComa with mild 3 atypia. The girl had an uneventful recovery, and after three years of follow-up, there were no signs of recurrence.

This case represents a rare instance of a benign PEComa, with a three-year follow-up, affecting the soft tissue in the knee region in a girl younger than 10 years. We report the management of this rare tumor whose clinical entity may range from benign atypia to aggressive malignancy, and for which there is limited literature regarding its natural history.

## Case presentation

A seven-year-old girl was referred to our pediatric orthopaedic department for the evaluation of her right knee. Her family had noticed a prominence beneath her patella for one month. Her family suggested a traumatic lesion; however, there was no clear history of injury. The girl participated in all daily activities without complaints or pain.

**Figure 1:**
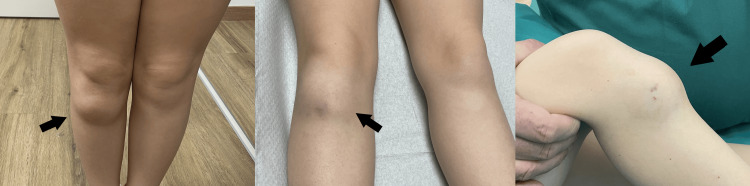
Prominence in the area of the tibial tubercle presenting as a painless mass (arrows).

Physical examination revealed a healthy child with a full range of flexion and extension of the right knee. A clear prominence was noted at the level of the tibial tubercle. The lesion was well encapsulated and firm, without discolorations or skin adhesions (Figure 1).

**Figure 2:**
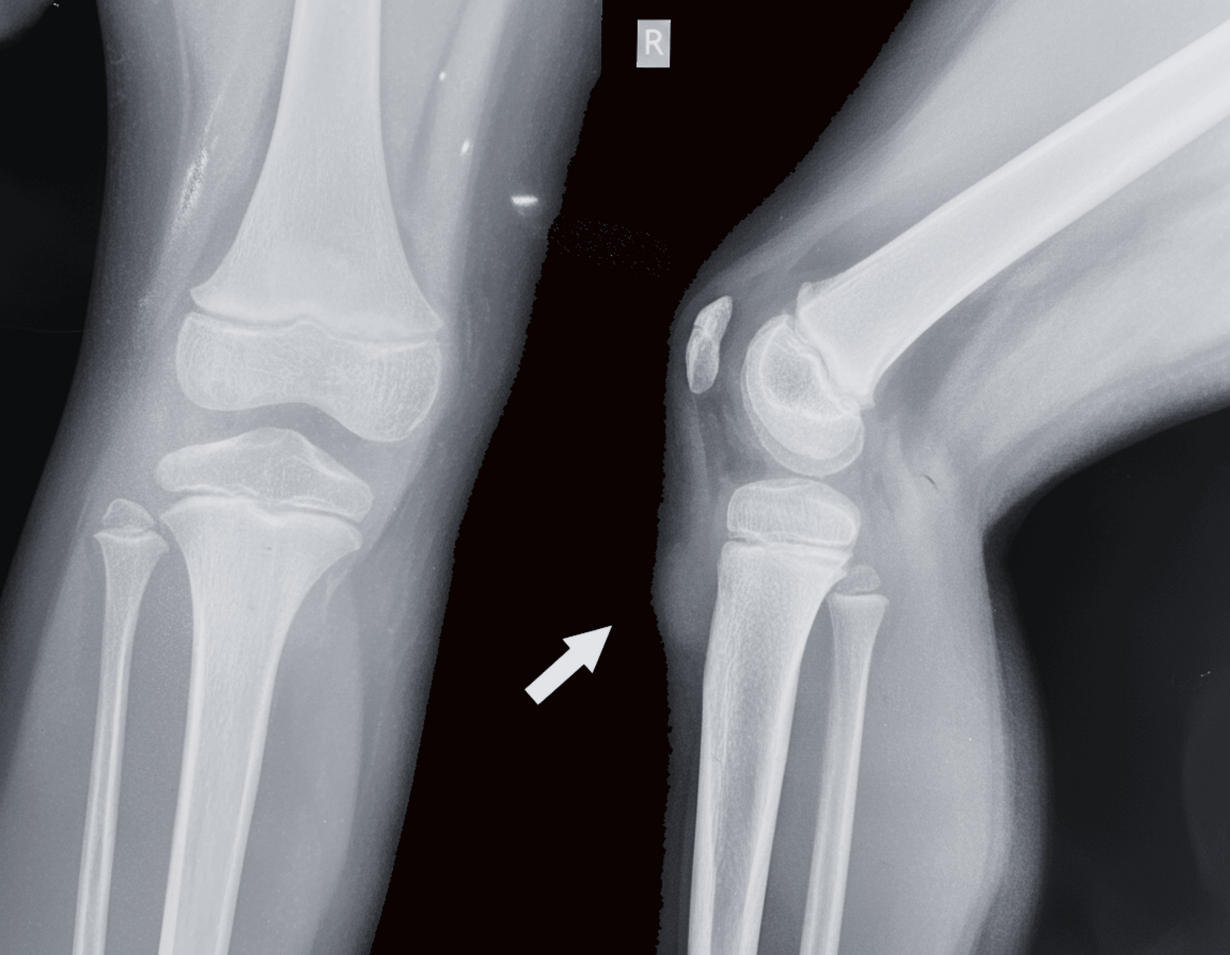
X-ray of the knee demonstrating a clear margin soft tissue enlargement, with intact bone and no periosteal reaction (arrow).

An X-ray of her knee, undertaken in her local hospital, showed a round, clear mass in the subcutaneous tissue just anterior to the tibial tubercle, with normal bone contouring and no evidence of periosteal reaction (Figure 2).

An MRI examination revealed a well-encapsulated mass measuring 4.1 cm in diameter, with several septa within the mass and clear margins. The mass was not connected to the underlying bone, and no signs of calcified tissue were reported.

**Figure 3:**
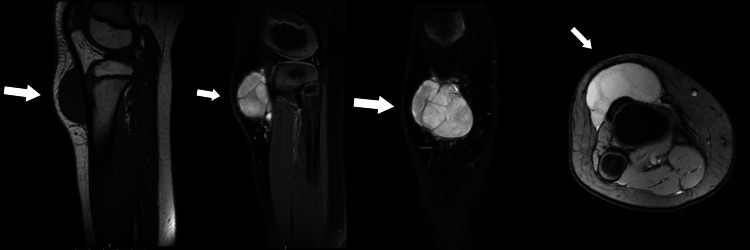
MRI images show an oval lesion on subcutaneous area in front of the tibial tubercle with increased signal intensity on T2-weighted images and low signal intensity on T1-weighted images. There are thin internal septa with smooth external contour and no bone involvement. There is absence of surrounding edema (arrows).

Fluid levels were not detected. The mass exhibited T1-weighted hypointensity and T2-weighted hyperintensity. Although these MRI findings are atypical of a simple lipoma, the presence of a firm mass with septa could indicate a lipoma. Vascular lesions and hematomas were included in the differential diagnosis (Figure 3).

**Figure 4:**
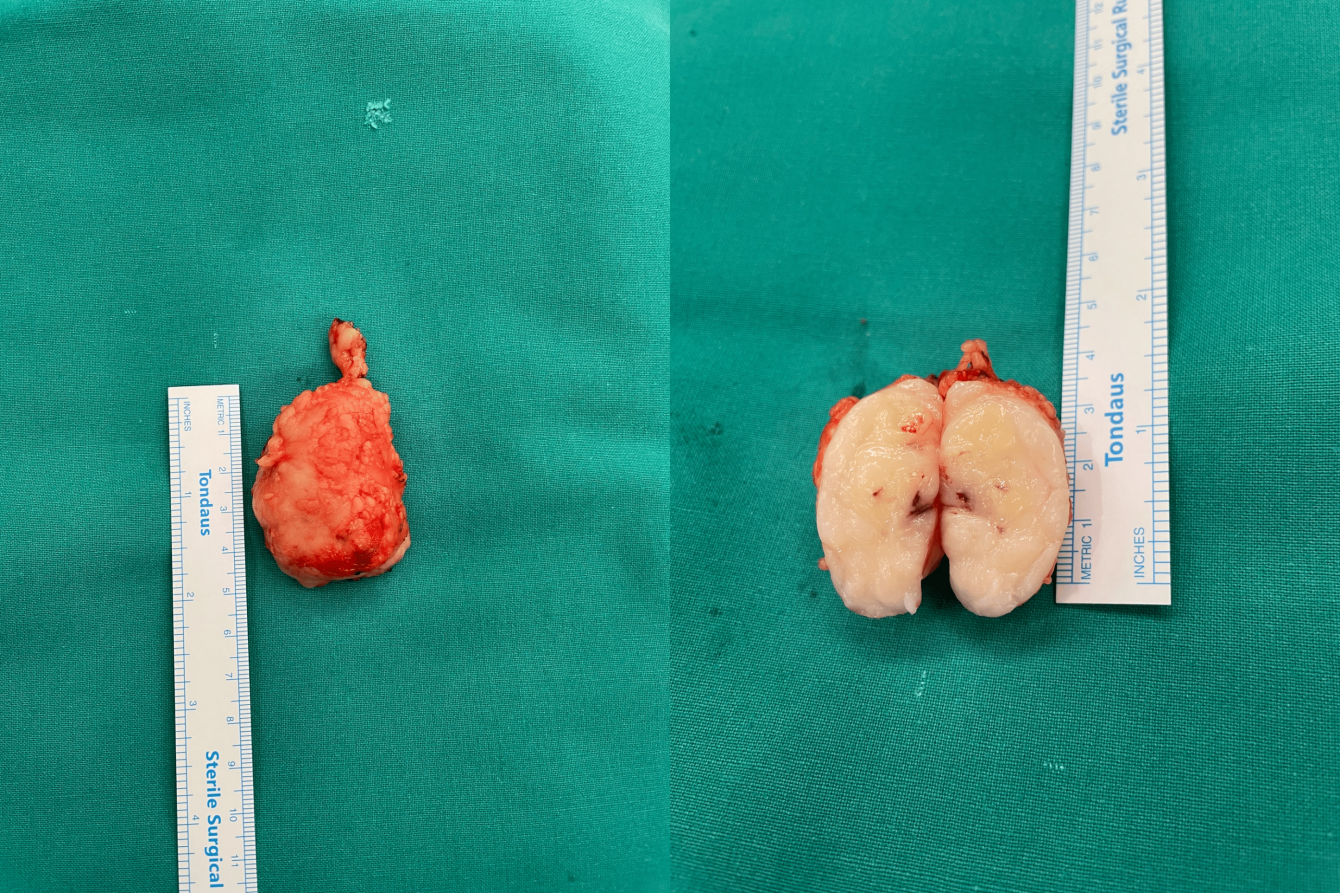
The mass is marginally 5 cm in diameter, appearing firm and elastic, giving the impression of a lipoma.

Following parental counselling, the patient underwent an excision biopsy. A well-encapsulated tumor was removed from the normal subcutaneous tissue. The mass was easily dissected from the surrounding tissue with clear margins and no evidence of increased vascularity or adherence to the fascia. Gross sectioning of the specimen initially reinforced the clinical impression of a benign lipomatous tumor (Figure 4).

Microscopic analysis of the surgical specimen revealed a tumor composed of large epithelioid cells arranged in a solid pattern and surrounded by a thin fibrous capsule.

The neoplastic cells were predominantly large and epithelioid, with a small proportion of spindle cells, and exhibited abundant, clear, and fine granular cytoplasm, vesicular nuclei with mild atypia, and rare mitotic activity. The tumor cells were arranged in solid aggregates, with numerous thin-walled vessels observed between them. Immunohistochemical (IHC) profiling showed that the tumor cells were positive for vimentin and caldesmon and focal positivity for desmin, while being negative for CK AE1/AE3, CD68, S100, CD34, SMA, HMB45, melan A, chromograninA, and synaptophysin.

**Figure 5:**
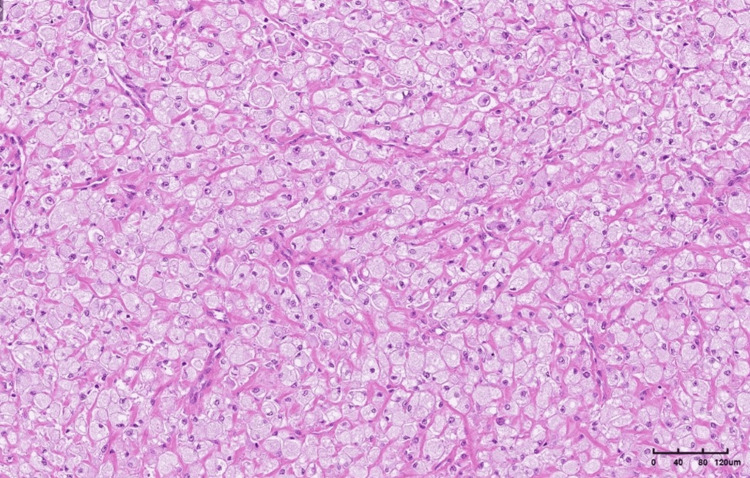
The tumor is composed of large epithelioid cells with compressed capillary vessels between them in a solid arrangement (hematoxylin and eosin (H&E) stain, ×10).

**Figure 6:**
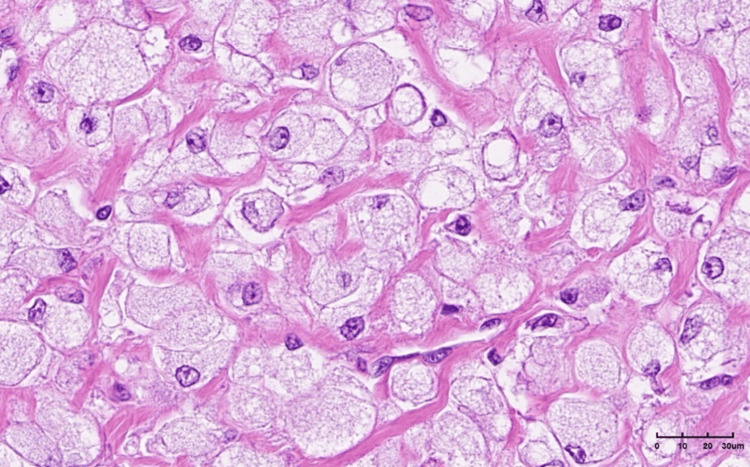
Epithelioid cells with clear, finely granular cytoplasm, with mild atypia and rare mitosis (hematoxylin and eosin (H&E) stain, ×40).

**Figure 7:**
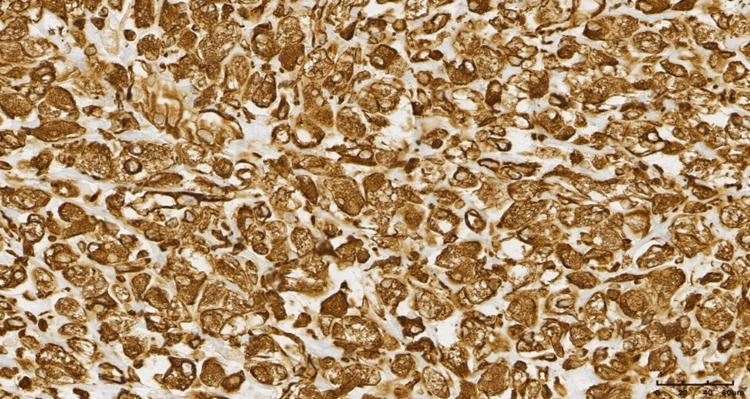
Immunohistochemical staining positive for vimentin (immunohistochemistry (IHC), ×20).

**Figure 8:**
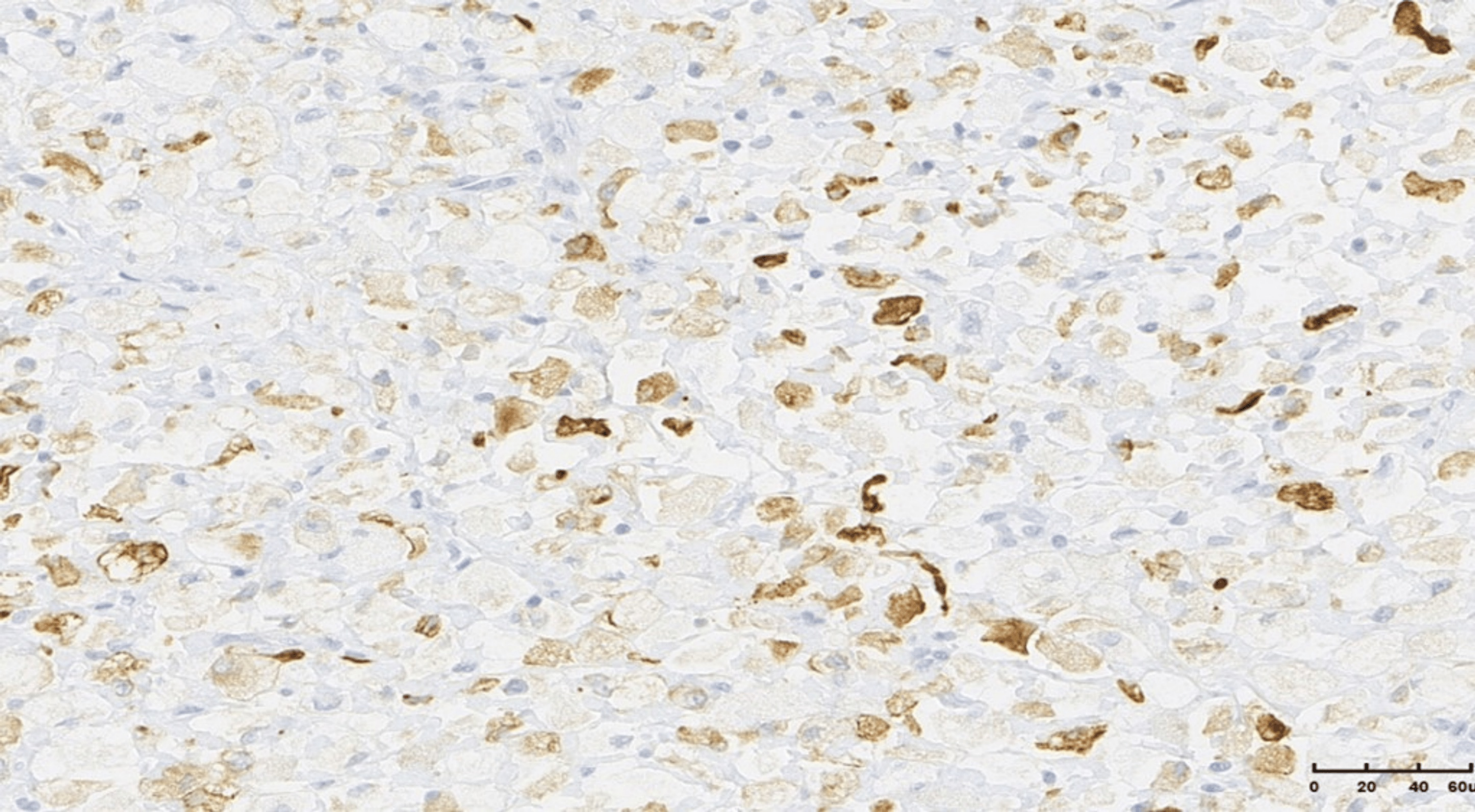
Focal positivity for desmin (immunohistochemistry (IHC), ×20).

**Figure 9:**
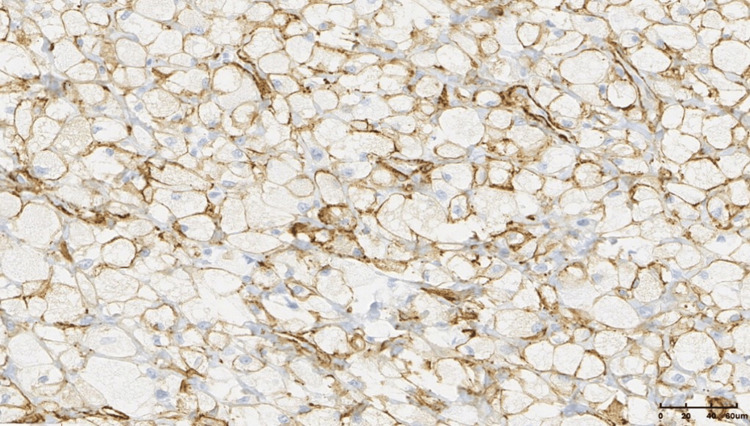
Positivity for h-caldesmon with membranous staining (immunohistochemistry (IHC), ×20).

Although the findings were not typical, the combination of histological picture with positivity for desmin was more consistent with a PEComa of the soft tissue with a low malignant potential (Figures 5-9).

The postoperative course was uneventful. Given the rarity of the diagnosis and the potential for late recurrence in PEComas, a rigorous surveillance schedule was implemented. The patient was monitored monthly for the first trimester, transitioning to semi-annual reviews thereafter. At the three-year follow-up mark, clinical and radiological assessments confirmed no evidence of local recurrence or systemic metastasis.

## Discussion

PEComas are a rare family of neoplasms of mesenchymal origin, initially proposed by Bonetti et al. [8,9]. In 2002, the World Health Organization introduced a new category of PEComas, defined as abnormal mesenchymal neoplasia characterized by perivascular epithelioid cells exhibiting distinctive histological and IHC features of both smooth muscle and melanocytic differentiation [10].

Primary PEComas are predominantly reported in adults, with the most frequent signs of involvement being the uterus, lung, kidneys, gastrointestinal tract, and liver [5,11]. Recent literature reviews have reported primary liver PEComas, where most patients were asymptomatic, with a palpable abdominal mass being the only sign of the disease. CT scans were the most common imaging method used for diagnosis, with definitive diagnosis being achieved through fine needle biopsy or laparoscopy in only 30% of patients [4]. An adrenal PEComa in a 63-year-old man was reported, highlighting the diagnostic challenges posed by the morphologic and immunophenotypic features of the neoplasm [12].

In a recent review by Caliò et al., renal PEComas have been discussed in detail, supported by histological, immune, and molecular findings [2]. Folpe et al. proposed a classification system in which the diagnosis of malignancy is determined by the presence of two or more high-risk factors: size greater than 5 cm, infiltrative growth pattern, high nuclear grade and cellularity, high mitotic rate, necrosis, and vascular invasion [13]. These patterns are primarily derived from gynecologic sources [14].

Garzon et al. compared the proposed classification system for predicting malignant versus non-malignant behaviour of uterine PEComas and found that the modified Folpe system was the most accurate [1,11]. According to Folpe classification, our case, with a size of 5 cm, encapsulated growth, mild atypia, rare mitotic activity, absence of necrosis and vascular invasion, is described as a benign PEComa.

PEComas are extremely rarely reported in children, with soft tissue involvement of the limbs being particularly uncommon. Vellaisamy et al. reported a well-encapsulated gray-white lesion of the right thigh in an 11-year-old girl. Contrast-enhanced computed tomography (CECT) was used for investigation and showed heterogeneous enhancement in the arterial phase, suggesting a possible diagnosis of hemangioma [7]. In our patient, the vascularity of the lesion was within normal limits. Alaggio et al. reported a malignant PEComa of the ligamentum teres in a two-year-old girl, characterized by local relapse after primary treatment with chemotherapy and surgery, and poor response to imatinib mesilate and temsirolimus. Further analyses confirmed that p70S6K expression was involved in the mTOR pathway. The girl was eventually treated with a debulking surgical procedure and is now alive with the disease, six years after its diagnosis [3].

PEComas primarily affecting bone are also rare [15]. Yamashita and Fletcher reported six patients aged between 35 and 74 years. Each of these patients presented an osteolytic destructive mass of the bone [16]. Röhrl et al. reported a malignant PEComa of the anterior part of the tibia in a 31-year- old female, who presented with a mass and weight loss. MRI showed a contrast-enhanced mass, and biopsy confirmed a malignant PEComa that was surgically removed along with a thin section of the tibia [17]. MRI findings are often nonspecific and not diagnostic for the lesion. In our case, MRI revealed T2-weighted hyperintensity, which lacked the pathognomonic features required to differentiate it from other soft tissue tumors [6,7].

Histological differential diagnoses for PEComas are broad and include alveolar soft part sarcoma, clear cell sarcoma, alveolar rhabdomyosarcoma, and metastatic carcinoma. Characteristic microscopic features, such as epithelioid and spindle cells surrounding thin-walled vessels with melanin pigment, can help the accurate diagnosis, further supported by appropriate immunostains. PEComas histologically express markers of melanocytic and smooth muscle differentiation. The rarity of mitosis and atypical cells indicates benign behavior of the tumor [7,11].

The pathogenesis of PEComas is multifactorial, including signalling pathway dysregulation, genetic alterations, and the influence of hormones and growth factors [5,18]. A proportion of PEComas have been reported to affect patients with TSC, a hereditary disease caused by mutations in the TSC1 and TSC2 genes, which lead to the constitutive activation of the mTOR signalling pathway, driving cell growth [11]. Ramezanpour et al. presented a case of a 22-year-old patient with TSC who presented an asymptomatic mass in the diaphysis of the left fibula. Despite the potential for progression, the patient declined surgical intervention. Serial MRI follow-up over time demonstrated only a minimal increase in tumor dimensions, suggesting a relatively indolent course in that specific skeletal location [19].

Pediatric presentation of TSC-associated PEComa can mimic benign fibrous lesions. Bajaj et al. described a four-year-old male child with TSC who presented with a firm mass on the anteromedial aspect of the right knee. The lesion on the MRI was well-circumscribed and markedly hypointense on all MRI sequences. The mass was surgically excised, and pathology confirmed a fibroma-like PEComa [6].

Having such a rare incidence, the optimal management and long-term surveillance of PEComas is often challenging. Surgical resection is the mainstay of their treatment, while adjuvant therapies, including chemotherapy or targeted therapy, may be considered in advanced or metastatic disease, although their effectiveness is limited. Surgical resection remains the best treatment for localized, benign PEComas [1,10]. For malignant or unresectable disease, chemotherapy and anti-angiogenic agents have shown limited effectiveness. Considering its pathogenesis, genetic therapy with mTOR inhibitors, such as rapamycin, may represent an effective treatment option for aggressive cases [10,11,18].

## Conclusions

PEComas are mesenchymal tumors that rarely affect the soft tissues of limbs in children. Radiological examinations have not revealed any specific characteristics of PEComas. Appropriate histological examination reveals spindled and epithelioid cells around vessels. IHC studies demonstrate features of both smooth muscle and melanocytic differentiation. The small tumor size of less than 5 cm and the absence of high-grade cellular atypia, necrosis, or significant mitotic activity in our patient supported a benign clinical course. Surgical treatment of the lesion in our patient was curative, with no signs of recurrence over a three-year period.
